# Editorial: Advances in the Immunology of Host Defense Peptide: Mechanisms and Applications of Antimicrobial Functions and Beyond

**DOI:** 10.3389/fimmu.2021.637641

**Published:** 2021-02-25

**Authors:** Thanh Kha Phan, Charles L. Bevins, Mark D. Hulett

**Affiliations:** ^1^Department of Biochemistry and Genetics, La Trobe Institute for Molecular Science, La Trobe University, Melbourne, VIC, Australia; ^2^Department of Microbiology and Immunology, School of Medicine, University of California, Davis, Davis, CA, United States

**Keywords:** immunomodulation, antimicrobial peptides, host defense peptides, antibiotic resistance, innate immunity

## Introduction

The term “antimicrobial peptides” (AMPs) classically refers to naturally occurring molecules that are made by most living organisms as key components of innate immunity that help shape the composition of colonizing micrbiota and provide a first line of defense against invading microbial pathogens. Although AMPS have diverse primary sequences, they are typically small in size, positively-charged, amphipathic, and contain α-helix and/or β-sheet rich structures often stabilized by disulfide bonds. AMPs can be constitutively expressed or induced by infectious or inflammatory stimuli, including proinflammatory cytokines and pathogen-associated molecular patterns. Since their discovery over a half century ago, research into AMPs has primarily focused on their direct antimicrobial activity, leading to them being touted as a promising molecular resource to tackle infectious diseases and antibiotic resistance. However, the direct microbicidal function of many AMPs may be limited under physiological and pathological conditions, due to a number of issues such as low local peptide concentration, suboptimal ion concentrations, physiological polyanions (e.g., mucins and glycosaminoglycans) and proteolytic degradation. More recently, a growing body of evidence suggests the biology and immunology of AMPs extends beyond the classical direct antimicrobial property. To highlight the multi-functionality and complexity of AMPs, in this Research Topic, we assembled a collection of original research and review articles that encompass recent advances in AMP research, with a particular emphasis on their alternative physiological and pathological functions. In doing so, we adopted a broader and more comprehensive term, “host defense peptides” (HDPs). New strategies to leverage HDPs for therapeutic uses, through exploitations of their multifaceted activities, are also highlighted.

## Immunomodulatory Function of HDPs

The ability of HDPs to mediate protection against microbial infection *in vivo*, under conditions that are often unfavorable for their direct antimicrobial actions, suggests that their immunomodulatory activities might be attributed to other functions. Indeed, HDPs have now been shown as potent immune effectors via multifunctional and multimodal orchestration of immune responses, including leukocyte chemotaxis, cytokine induction, angiogenesis and phagocytosis, modulation of leukocyte development and survival, as well as neutralization of pathogen-associated molecular patterns (PAMPs). HDPs also provide a link between innate and adaptive immunity by mediating chemotactic recruitment of antigen presenting cells and T lymphocytes, stimulating maturation of dendritic cell (iDC) maturation, and providing adjuvant-like functions. For example, proinflammatory HDPs, including iron-sequestering lipocalin 2 (Lcn2) can amplify antimicrobial responses to enhance pathogen killing and clearance. Lcn2-deficient mice, which result in reduced neutrophil recruitment and impaired cytokine production in macrophages, are more susceptible to *Escherichia coli* infections (Wang et al.). In contrast, Koeninger et al. demonstrated for the first time that systemically administered human β-defensin 2 (HBD-2, also know as DEFB2) suppresses inflammation induced by bacterial PAMPs including lipopolysaccharides (LPS), and in experimental colitis mouse models through chemokine receptor 2 (CCR2) targeting. The anti-inflammatory functions of HBD-2, and likely other HDPs, are required for controlling bacteria killing-associated inflammation and maintaining immune homeostasis, thus limiting unwanted inflammation-induced tissue damage. Similarly, cathelicidins attenuate LPS-induced Toll-like receptor-dependent inflammation through direct LPS-cathelicidin interaction. However, they can also bind to and stabilize bacterial DNAs and RNAs, promoting nucleic acid-sensing TLR activation. In their review, Scheenstra et al. provided a comprehensive discussion of these contrasting cathelicidin-mediated TLR modulations in reducing pathogen burdens during infection and resolving excessive inflammatory responses. From these articles, we can appreciate that it is not always straightforward to classify HDPs as either pro-inflammatory or anti-inflammatory. Instead, it may be appropriate to define them as inflammation regulatory molecules that balance host inflammatory responses and sustain immune homeostasis.

## Microbiota Regulation and Tissue Homeostasis by HDPs

Beyond their involvement in innate defense against pathogens, recent studies have uncovered the importance of HDPs in tissue homeostasis, dictated not only by their canonical antimicrobial function but also by their immunomodulatory properties. Notably, within protective epithelial barriers, it is clear that HDPs are critical, not only in defending against pathogenic pathogens, but also in regulating commensal microbiota as well. This microbiota balance within epithelial tissues is key for heathy epithelial homeostasis and immunity (Coates et al.). To this end, colonic HDPs [cathelicidins, β-defensins, regenerating islet-derived protein III (Reg3) and resistin-like molecules (Relm)] form an interactive network, as proposed by Blyth et al. and function synergistically and multimodally to prevent enteropathogenic colonization. Puértolas-Balint and Schroeder reviewed the complex interactions between diet, commensal microbiota and HDPs in gut homeostasis and diet-associated inflammatory diseases. They document that nutrient-sensing signaling mediators are involved in diet-associated modulation of HDPs, while immune mediators and effectors shape microbe-dependent HDP regulation. Furthermore, intestinal HDPs display unique mechanisms of action and spectrum of activity to regulate microbiota composition, evidently beyond direct antimicrobial activity. Of note, HDP-mediated control of gut microbiota is dependent on localization and the specific regulatory mechanisms of expression and activation. In support of this, in their original study, Nakamura et al. showed that Paneth cell-secreted α-defensins are topographically regulated from duodenum to ileum. Their findings of ex-germ free-dependent regulation of α defensins also suggest that the intestinal microbiota may partially affect HDP activity. Nevertheless, it remains to be determined precisely how microbes and certain dietary components can signal to the epithelium and mediate HDP function, as well as how HDPs mechanistically modulate gut microbiota through immunomodulation. Beyond human epithelial barrier, Le Bloa et al. discovered type IIa crustin, as a novel HDP in the deep-sea shrimp *Rimicaris exoculate*. This investigation uncovered an expression pattern of type IIa crustin that is spatio-temporally correlated with ectosymbiotic colonization through different life stages. These findings further signify the importance of HDPs in the establishment of vital and heathy host-symbiotic microbiota through their multifaceted functions.

## HDPs in Diseases and Chronic Inflammatory Conditions

As our understanding of molecular and cellular mechanisms underpinning HDP immunomodulation improves, the roles of HDPs in various diseases and chronic inflammatory conditions are drawing an important focus. This Research Topic features several reviews on the multitude of pathological outcomes associated with dysregulation of cathelicidins and defensins, including skin disorders (e.g., psoriasis, atopic dermatitis), inflammatory bowel diseases (e.g., ulcerative colitis, Crohn's disease), autoimmune diseases, lung disorders (e.g., cystic fibrosis, chronic obstructive pulmonary disease, asthma), and tumorigenesis (Alford et al., Liang and Diana, Shelley et al., Wehkamp and Stange, Xu and Lu). These review articles collectively provide a thorough overview of HDPs' functions in pathology and converge to highlight the “double-edge sword” nature of HDP-mediated immunomodulatory responses. Of note, Lee et al. highlighted the unexpected similarities between Aβ amyloid aggregates and HDP protofibrils, hence posing new perspectives for HDP-associated pathogenesis. Intriguingly, by using ecogenetic analyses, Hanson et al. described an independent loss of immunologically costly and deleterious HDPs, such as diptericin, in true fly lineages in the absence of microbial challenges. The complexity of HDP-associated pathogenesis is further complicated by the heterogeneity of HDP (dys)regulation. In an original research contribution, Hemshekhar et al. showed that murine cathelicidin (CRAMP) and calprotectin (S100A8 and S100A9) are differentially regulated in the local tissues (joint and lung, respectively) in two immunopathologically-related mouse models of inflammatory arthritis and airway inflammation. Together, these reviews and research studies highlight that host immunity needs to strike a delicate balance in HDP regulation, to mediate an antimicrobial defense response on the one hand, whilst avoiding HDP-induced pathological perturbance on the other.

## Clinical Applications for HDPs: Opportunities and Challenges

Insights into HDP mechanisms and functions have opened new and exciting therapeutic avenues, and have provided novel platforms for therapeutic design. In this Research Topic, potential clinical uses for HDPs are extensively discussed in various disease conditions, ranging from multidrug-resistant bacterial infection (Bergman et al.), fungal infection (Mercer and O'Neil), and cancer (Xu and Lu) to autoimmune disorders (Liang and Diana) and chronic inflammatory diseases (Alford et al., Koeninger et al., Shelley et al., Wehkamp and Stange). The broad-spectrum antimicrobial effects of HDPs have long-inspired the development of these peptides for treatment of infectious diseases. In addition, their actions on bacterial biofilms may benefit rational engineering and designs to tackle the biofilm-associated resistance against host immunity and antibiotics. For instance, Parducho et al. reported the antibiofilm activity of HBD-2 against *Pseudomonas aeruginosa* and *Acinetobacter baumannii*, is independent of biofilm regulatory pathways and instead mediated via induced alteration of biofilm integrity. It should also be noted that in the non-infection settings, the immunomodulatory functions of HDPs can also potentially be exploited to restore the balance to dysregulated immune responses that contribute to disease pathologies.

However, there are several challenging obstacles for HDP applications, notably their potential toxicity, proteolytic instability, salt intolerance and low *in vivo* efficacies. In an attempt to address the gap between bench-to-bedside translation of HDP-based therapies, Ting et al. highlighted different strategies in designing and translating the therapeutic potentials of HDPs, such as novel HDP synthesis, sequence optimization, cyclisation, N–/C–terminal modification and hybridization. Significantly, the authors also proposed a strategic pipeline for the developmental pathway of HDP-based therapeutics, from novel HDP prototyping to conducting preclinical studies to optimally inform further clinical trials. Consistent with these outlined strategies, Hernandez et al. demonstrated cyclic peptide [R_4_W_4_] with an improved antimycobacterial effect. Learning from the evolution of invertebrate big defensins toward β-defensin, Gerdol et al. also suggested to ameliorate HDPs' salt intolerance through N-terminal hydrophobicity. Ehmann et al. enhanced the potency of human neutrophil α-defensin four against multidrug resistant bacteria through truncation, N-terminal acetylation and C-terminal amidation. Additionally, Rodríguez-Rojas et al. found that *Staphylococcus aureus* is highly susceptible to toxic nucleoside analogs when exposed to pexiganan, and based upon this finding, proposed HDP-based combinatorial therapy for infectious diseases. Furthermore, Ting et al. provided a stimulating discussion on the use of artificial intelligence technology with machine learning algorithms as a tool to search for HDP sequences with desirable efficacy. Indeed, the BACIIα algorithm, a tensor search protocol, was used by Yount et al. to identify >700 putative new prokaryotic bacteriocins, which display potent *in vitro* antimicrobial effect against a wide range of human pathogens. Similarly, Cho et al. developed a digitized framework for *in silico* HDP identification and characterization, by which 19 novel cathelicidins from the gray short-tailed opossum were revealed and experimentally validated for their antibacterial and anti-West Nile virus activities. Undoubtedly, machine learning will help revolutionize future HDP discovery, which should not only shed light upon HDP biology in understudied organisms (such as plant HDPs as argued by Petre), but also expand the HDP arsenal for therapeutic exploitation.

## Concluding Remarks

Together, the papers in this collection highlight and add new knowledge on the multifaceted function and importance of HDPs in contributing to the shape and complex nature of host immunity, as well as in regulating health and mediating disease progression. As our knowledge of HDP biology expands, the repertoire of HDP functions, particularly direct antimicrobial activity and immunomodulation, demonstratively opens the door for novel therapeutic development for treating infectious diseases, as well as inflammatory conditions. Nevertheless, while the classical microbicidal property of HDPs has been extensively studied, the research journey has only just begun for their non-antimicrobial properties. Forsaking the classical term “antimicrobial peptides” and embracing “host defense peptides” can not only properly acknowledge the multi-functionality and complexity of HDPs, but may also imprint the currently underexplored activity landscapes and enshrouded therapeutic potential of HDPs. We would like to take this opportunity to thank the authors for their invaluable contributions to this Research Topics, as well as all the reviewers for their time and constructive inputs.

## Author Contributions

TKP wrote the manuscript. CB and MH critically revised and approved the final version of the manuscript. All authors conceived the outline of the manuscript.

## Conflict of Interest

The authors declare that the research was conducted in the absence of any commercial or financial relationships that could be construed as a potential conflict of interest.

